# A Toolkit for Detecting Fallacious Calls for Papers from Potential Predatory Journals

**DOI:** 10.34172/apb.2023.068

**Published:** 2023-01-23

**Authors:** Mehdi Dadkhah, Abdul Majed Raja, Aamir Raoof Memon, Glenn Borchardt, Prema Nedungadi, Khaled Abu-Eteen, Raghu Raman

**Affiliations:** ^1^Amrita School of Engineering, Amrita Vishwa Vidyapeetham, Amritapuri, Kerala, India.; ^2^Technology forecasting department, SnowaTec Technology center and Innovation Factory, Entekhab Industrial Group, Isfahan, Iran.; ^3^Data Scientist, Atlassian, Bengaluru, Karnataka, India.; ^4^Institute for Health and Sport, Victoria University, Melbourne, Australia.; ^5^Progressive Science Institute, 1966 TICE VALLEY BLVD #172, WALNUT CREEK, CA 94595-2203, USA.; ^6^Amrita School of Computing, Amrita Vishwa Vidyapeetham, Amritapuri, Kerala, India.; ^7^The Hashemite University, P.O. Box 330127- Zarqa 13115, Jordan.; ^8^Amrita School of Business, Amrita Vishwa Vidyapeetham, Amritapuri, Kerala, India.; ^9^Amrita School of Engineering, Amrita Vishwa Vidyapeetham, Amaravati, Andhra Pradesh, India.

**Keywords:** Predatory journal, Sentiment analysis, Academic ethics, Journal publishing, Calls for papers, Data science

## Abstract

**Purpose:**

Flattering emails are crucial in tempting authors to submit papers to predatory journals. Although there is ample literature regarding the questionable practices of predatory journals, the nature and detection of spam emails need more attention. Current research provides insight into fallacious calls for papers from potential predatory journals and develops a toolkit in this regard.

**Methods:**

In this study, we analyzed three datasets of calls for papers from potential predatory journals and legitimate journals using a text mining approach and R programming language.

**Results:**

Overall, most potential predatory journals use similar language and templates in their calls for papers. Importantly, these journals praise themselves in glorious terms involving positive words that may be rarely seen in emails from legitimate journals. Based on these findings, we developed a lexicon for detecting unsolicited calls for papers from potential predatory journals.

**Conclusion:**

We conclude that calls for papers from potential predatory journals and legitimate journals are different, and it can help to distinguish them. By providing an educational plan and easily usable tools, we can deal with predatory journals better than previously.

## Introduction

 The term “predatory journals” was coined in 2010 to reflect journals for whom financial gains are more important than quality and ethics in publishing.^1-3^ There was a list, famous as “Beall’s list,” of potential predatory journals and publishers maintained for several years; however, the list was defunct in 2017 and later faced criticism from different scholars.^4-6^ Predatory journals do not have a peer review and are therefore considered a threat to the integrity of science because the published findings can lead to the propagation of erroneous or unverified results with a potential for severe consequences.^7^ Under the temptation to earn more money, predatory journals accept almost all papers irrespective of the quality of their content.^1-3^

 One of the characteristic features of predatory journals is sending unsolicited call for papers (CFP) to different researchers irrespective of their field of interest.^1,8-10^ Several researchers have studied whether a journal is predatory based on the CFPs. For example, Moher and Srivastava collected CFPs received over one year and concluded that 79% of CFPs were from predatory journals appearing on Beall’s list.^11^ Some researchers have suggested that prospective authors only consider CFP from well-established journals.^12^ Mercier et al analyzed 237 CFPs from potential predatory journals received over 12 months and found that only 13.5% disclosed their publication fee, 70.5% stated that they accept all types of articles, 69.6% mentioned a deadline for publishing the papers, 34.1% claimed to have a peer review process, and 9.3% used misleading metrics.^13^ Memon analyzed spam emails received over 18 months and reported common features of predatory journals which were identified from their CFPs, including the use of attractive names, fake and bogus metrics, claims for indexation in well-known citation databases, questionable review practices, presence of article processing charges, presence on Beall’s list and several others.^14^ Lewinski and Oermann analyzed CFPs from 206 predatory journals and found that such emails use flattering language, have tight deadlines, and use awkward phrases.^15^ Sureda-Negre and colleagues had 210 spam emails received over three months in educational science. They conclude that half of CFPs are not in the field of the recipient, and half of the predatory journals’ domains do not have trustworthy security levels.^16^

 It should be noted that legitimate publishers also send CFPs emails to authors. However, such emails are mostly received after subscription, solicited, or have an academic writing style and content.^10^ However, some low-quality legitimate journals might send unsolicited CFPs to researchers creating a grey area between legitimate and predatory journals.^10^

 It is evident that some research examining the CFPs from potential predatory journals was conducted previously. However, these journals are likely to change their fraudulent techniques in response to the growing research to avoid such journals. In addition, to the best possible we know, there are no tools to detect unsolicited calls for papers from potential predatory journals. Therefore, the aim of this research was 1) to determine the difference between CFPs from predatory journals vs. legitimate journals, 2) to detect changes in CFPs from predatory journals during recent years, 3) and to develop a tool for detecting unsolicited CFPs.

*RQ 1-Have there been significant changes in CFPs from predatory journals during recent years*?

*RQ 2-Is there still a difference between CFPs from predatory journals and those from legitimate journals*?

*RQ 3-If so, how can we detect CFPs from predatory journals now*?

*RQ 4-Is it possible to develop a tool for detecting unsolicited calls for papers*?

## Methods

 The current study uses three datasets: 1) 104 unsolicited (i.e., spam) emails received as CFPs by authors of this study during the period from April 15, 2020, to May 15, 2020 (dataset 1); 2) 138 CFP emails sent by recognized genuine journals/conferences or identified from http://www.call4paper.com during the same previous period (dataset 2); 3) another dataset shaped by using 160 unsolicited CFPs from potential predatory journals and 190 legitimate CFPs (dataset 3). When a journal sent multiple spam with minor or major changes, we kept all of them.

 Regarding RQ 1, we needed to understand the similarities and dissimilarities in the potential predatory CFPs. In this regard, we used a document clustering algorithm based on their similarities. This algorithm includes a machine learning subroutine that simplifies the clustering process. In this research, each email plays the role of a document. This method helped us detect predatory journals that used similar words in their CFPs. Also, it helped identify predatory journals that used templates characteristic of a particular publisher. The idea behind clustering is that data from documents belonging to a specific cluster should have a minimum distance.

 In contrast, data from documents belonging to different clusters should have a maximum distance. We use cosine distance to cluster documents. Also, the number of clusters has been set to ten based on trial and error to shape the best fitting of documents to clusters. To perform such an analysis, we followed tutorials on document clustering,^17-19^ with editions and adding new codes.

 To answer RQ 2 and RQ 3, To understand the main theme of each CFP in terms of perception, we implemented emotional analysis and calculated the sentiment score for each CFP email.^20^ We used the vignette by Feuerriegel and Proellochs^21^ to calculate the sentiment behind each CFP. We analyzed the emotional perception for dataset 1. We then compared the results of emotional perception between legitimate (dataset 2) and potential predatory CFPs (dataset 1).

 Finally, by considering RQ 4, a tool was developed to detect potentially predatory CFPs. We shaped a Lexicon to identify keywords used by supposedly predatory journals. These keywords were identified by analyzing 160 unsolicited CFPs from potential predatory journals and 190 legitimate CFPs (dataset 3). We developed an online tool using R shiny.^22^ Any researcher can now analyze any CFP without special technical knowledge. We tested this tool by using a sample of CFPs to understand how tool truly works.

**Figure 1:**
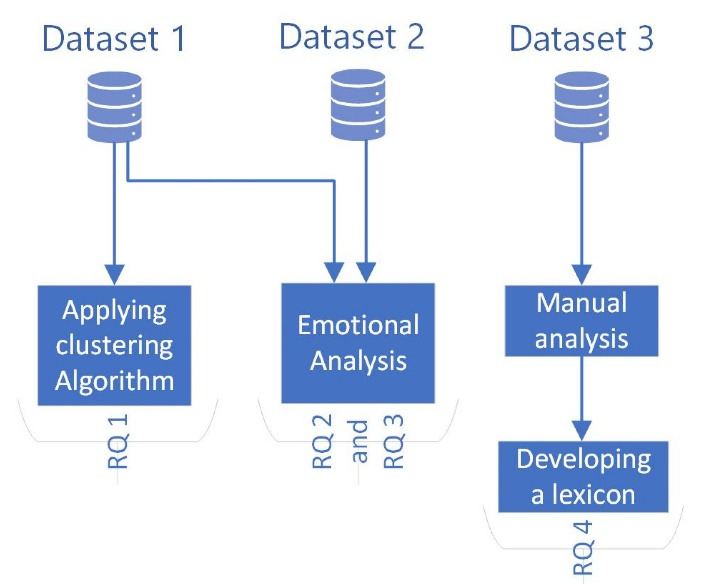
summary of the research process

 The algorithm and analyses were implemented using R^23^ programing language and its packages, including tools, data.table, readtext, tidytext, tm, proxy, SentimentAnalysis, ggplot2, wordcloud, shiny, tidyverse, shinythemes, shinyalert, and shinyWidgets.^24-37^ The statistical output was presented as figures (i.e., word cloud, clusters). The codes are available as Supplementary file 1. Figure 1 shows a summary of the research process.

## Results

 By checking the 104 spam emails (dataset 1), we detected 18 redundant emails, removing those identical to other emails. These spam emails were analyzed, extracting the name of publishers or journals from each spam email. Some spam emails mentioned a single journal and others mentioned a publisher. We identified a total of 74 journals and publishers. In some cases, there was a CFP from a predatory journal in one email and a CFP from its publisher in another. In such cases, we included a single journal and publisher in our list. We used Beall’s 2017 list of single potential predatory journals (https://web.archive.org/web/20170111172309/https://scholarlyoa.com/individual-journals/) and potential predatory publishers (https://web.archive.org/web/20170111172306/https://scholarlyoa.com/publishers/) as well as its updated list (https://beallslist.weebly.com). We did not limit ourselves to these lists and examined the journals’ quality. A total of 35 journals/publishers were available on Beall’s list.

**Figure 2:**
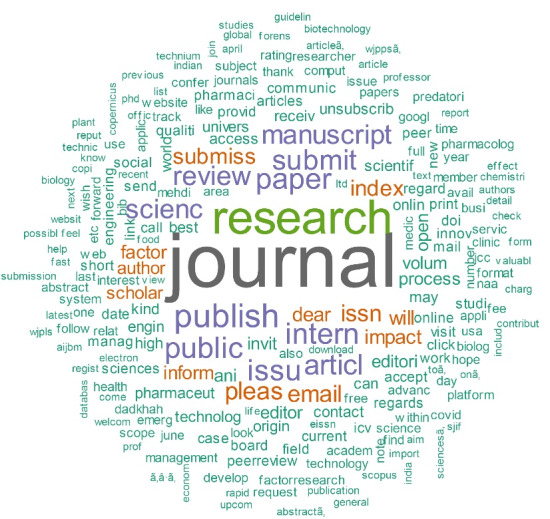
Word cloud of CFP from potential predatory journals (dataset 1)

 We analyzed these journals, besides other journals and publishers, and could identify predatory practices in most of them. So, our dataset mainly contains CFP from potential predatory journals or publishers or journals/publishers that follow questionable and predatory practices. We name these journals/publishers as the “possible/potential predatory” because there are critics to Beall’s list. In addition, we tried to examine each journal in terms of quality and detected questionable practices. Figure 2 shows the word cloud for the emails. This word cloud shows the most frequent words in the CFPs. The size of words shows their frequency.

**Figure 3:**
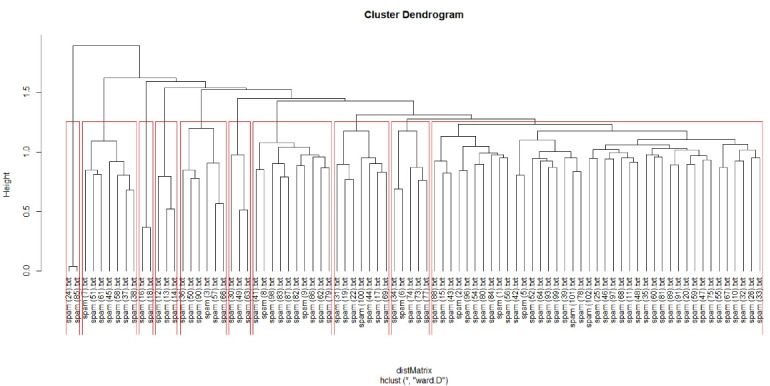
Clusters of CFPs based on their similarity (dataset 1)

 We applied the clustering algorithm to our data (dataset 1/content of each spam email). Based on the analysis of 86 spam emails originating from known predatory journals, we detected 10 clusters (Figure 3).

 Cluster 1 (on the extreme left) consisted of two spam emails from the same journal. Although these two emails were similar, the editor offered a discount for publication charges in one of them. This is a trick that predatory journals often use to encourage novice or inexperienced authors to submit papers (this practice is not unique to predatory journals because legitimate open-access journals also may provide a discount for their open-access fee).

 Cluster 2 included spam emails containing CFPs from predatory pharmacological journals. In these spam emails, an invitation for editorial board membership was included. Previous research shows that predatory journals are searching for new editors to increase their chances of receiving manuscripts from prospective authors who might be impressed by a prestigious editorial board.^37^

 Cluster 3 included two similar spam emails sent by a publisher for two different journals that used similar templates for all their spam emails. They only changed the name of the journals in each spam email.

 Cluster 4 included three spams from two different journals. Even though the websites of these two journals had different domains, their design was very similar. Launching a separate website for a predatory journal prevents bulk emailing detection that might label them as ‘predatory.’ Advanced predators often use different email templates to decrease the chance of being detected and labeled as ‘predatory.’

 Cluster 5 contains CFPs that are related to biology and chemistry journals. In these categories, similar templates were used for some CFPs, although their seemingly similar websites used different domain names.

 Cluster 6 included predatory publishers sending an identical email for all of their journals instead of sending a separate email for each.

 Cluster 7 contained journals in the field of forensic science or surgery. An interesting observation in the CFPs of this cluster was that it mentioned COVID-19. This predatory journal stated that they publish various types of papers on COVID-19. Memon and Rathore^38^ warn about the unfortunate and likely publication of some valuable COVID-19 papers in predatory journals.

 In cluster 8, predatory journals specializing in the business sent potential authors the table of contents of their journals and invited them to publish. They listed COVID-19 papers in the table of contents to receive papers related to COVID-19.

 Cluster 9 has CFPs from various fields including multidisciplinary science, engineering, agriculture, etc.

 Cluster 10, the biggest cluster, had spam from journals in various fields. This large cluster indicates that most predatory journals (at least in our sample) used similar words and structures to encourage authors to submit papers.

 Clusters 9 and 10 show spam emails that are not located in previous clusters and contain various fields. But, this variation in the fields can be separated based on the words in the CFPs into two different clusters.

**Figure 4:**
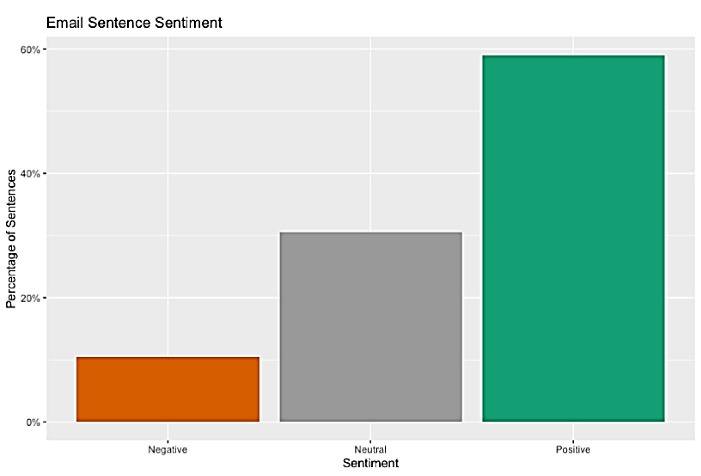
Distribution for the percentage of sentiments (negative, neutral, positive) in the language used in CFPs from potential predatory journals

 The sentiment analysis on dataset 1 identified three CFPs’ perceptions: negative, neutral, and positive (Figure 4). Most of the sentences in CFPs reflected a positive sentiment to encourage authors to submit papers. For example, there were claims that the journals are indexed in a reputable database, have a high impact factor (by referring to misleading metrics), offer fast peer-review, and have a quick or short time from manuscript submission to publication.^39,40^

**Figure 5:**
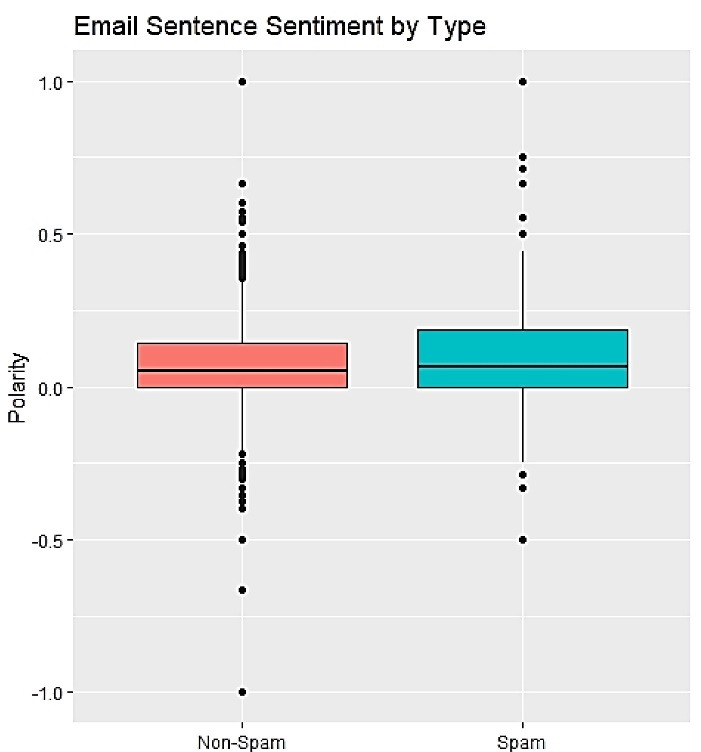
Comparing legitimate and predatory CFP sentiment in the form of polarity

 We compared legitimate (dataset 2) and potential predatory CFPs (dataset 1) about sentiment in the form of polarity. Spam emails were significantly more positive than solicited emails, as established by the t-test. Polarity can range from -1 (negative sentiment) to 1 (positive sentiment); a 95% CI of the difference between these groups is [-0.054, -0.024]. Both email classes gravitate toward positive language, although spam emails tend to be more ‘over the top’ with their word choice. Figure 5 compares legitimate and predatory CFP sentiment in the form of polarity.

**Figure 6:**
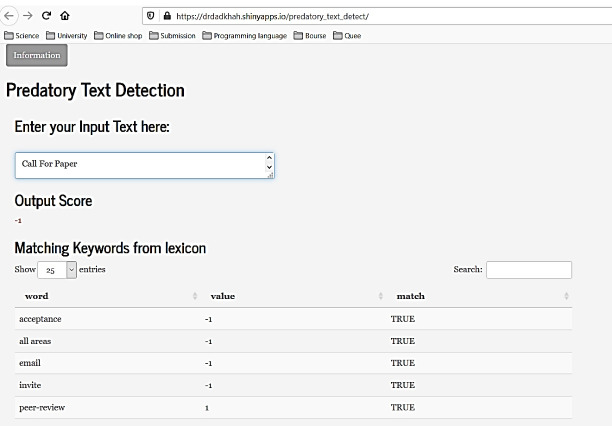
A tool for detecting predatory CFPs

 Based on mentioned findings, we conclude that potential predatory journals use specific terms in their CFP. Also, the predatory designation is not always clear—a journal may follow some predatory practices but avoid the more obvious ones. A study by Dadkhah and Bianciardi^41^ and Memon^10^ confirms this claim. As mentioned, predatory journals project an especially positive sentiment toward potential victims. Also, they usually choose similar keywords in their calls for papers. So, it is possible to detect predatory journals’ CFPs by analyzing the keywords in their CFPs. Therefore, we decided to develop a lexicon entitled “predatory lexicon” for the R tool. This lexicon contains words with two polarities: predatory or legitimate. We analyzed 160 unsolicited CFPs from potential predatory journals and 190 legitimate CFPs (dataset 3) to prepare a polarity lexicon containing 150 keywords and phrases. Now each CFP can be analyzed about this lexicon to understand its polarity and determine how likely the CFP might be from a possible predatory journal. We developed an online tool using R shiny.^22^ Any researcher can now analyze any CFP without special technical knowledge. The tool calculates a score for each email, and if it tends to be a predatory CFP, it shows “-1”; if it tends to be a legitimate CFP, it shows “1”. It also lists all the keywords or phrases used to design an email as predatory or legitimate (Figure 6). As mentioned, being predatory is a fuzzy term—a journal may be fully predatory or maybe only follow a few predatory practices. The tool indicates the relative degree to which a particular CFP has characteristics of a potential predatory journal.

Table 1 shows calculated scores for a sample of journals’ CFPs. These samples have been collected from the authors’ email (for potential predatory CFPs) and selected CFPs in http://www.wikicfp.com/cfp/ (For legitimate CFPs). Because predatory is a fuzzy term, some CFPs have many predatory words, and some have many legitimate words. Based on our approach, potential predatory CFPs usually contain more predatory keywords than legitimate keywords. In legitimate CFPs, there usually are fewer predatory keywords and more legitimate keywords. When the designation is close or doubtful, prospective authors need to investigate the journal being considered further. Also, predators will likely use our tool to prepare CFPs with a lower predatory theme. However, even if they use the tool, they are unlikely to remove all words necessary for their deception. Note that the CFPs of some legitimate journals are similar to those of predatory journals, causing our tool to label those CFPs as predatory by mistake. That merely indicates that such journals are suspicious, though not predatory. Their CFPs look like predatory ones. We recommend that journal editors use our tool to check their email content before broadcasting.

**Table 1:** Test results of the tool for the sample of CFPs emails

**Sample ID**	**Number of identified keywords from a potential predatory journal**	**Number of identified keywords from a potential legitimate journal**	**Real CFP category**	**Description based on the present approach**
1	3	1	Predatory	This is a predatory CFP
2	3	0	Predatory	This is a predatory CFP
3	3	2	Predatory	This is a predatory CFP
4	4	1	Predatory	This is a predatory CFP
5	1	0	Predatory	This CFP looks like a predatory CFP
6	5	0	Predatory	This is a predatory CFP
7	3	1	Predatory	This is a predatory CFP
8	3	2	Predatory	This CFP looks like a predatory CFP
9	3	1	Predatory	This is a predatory CFP
10	6	0	Predatory	This is a predatory CFP
11	4	1	Predatory	This is a predatory CFP
12	5	2	Predatory	This is a predatory CFP
13	6	1	Predatory	This is a predatory CFP
14	2	1	Predatory	This CFP looks like a predatory CFP
15	1	2	Legitimate	This is a legitimate CFP
16	1	1	Legitimate	This CFP looks like a predatory CFP
17	2	1	Legitimate	This CFP looks like a predatory CFP
18	1	2	Legitimate	This is a legitimate CFP
19	2	0	Legitimate	This legitimate CFP has been designed like predatory CFP.
20	3	2	Legitimate	This CFP looks like a predatory CFP
21	5	2	Legitimate	This legitimate CFP has been designed like predatory CFP.
22	1	0	Legitimate	This CFP looks like a predatory CFP
23	0	1	Legitimate	This is a legitimate CFP

## Discussion

 Cited research in introduction sections^10-15^ analyzed such spam two or four years ago. Our analysis of current spam does not disclose significant changes except for the opportunistic COVID-19 inclusion. Therefore, like the infamous “Nigerian Prince” spam, predators did not implement significant changes in their emails (answer to RQ 1). However, we anticipate predatory journals will become more sophisticated by trying to look more legitimate in the future. This highlights the need for training and efforts to increase public awareness.

 By considering Figure 5, it is clear that potential predatory journals usually try to make positive sentiments and perceptions for potential authors about their journals, mostly avoiding anything that would sound negative (answer to RQ 2).

Table 2 illustrates features that can help distinguish CFPs from potential predatory journals from legitimate journals (answer to RQ 3). We extracted this feature by comparing potential predatory journals’ CFPs (dataset 1) with those of legitimate journals (dataset 2) by considering Figure 3. As in each cluster, emails usually are similar, so these similarities can help to identify features for CFPs from predatory journals. Interestingly, these features are especially similar to those found by others.^13-15^

**Table 2:** Features identifying CFPs from predatory journals

**Number**	**Questions**	**Answers that increase the chance of being a predatory journal CFP**
1	Have you subscribed to receive such an invitation?	No
2	Is there any misleading metric or questionable indexing service mentioned in the CFP?	Yes
3	Does the journal have an extremely broad scope?	Yes
4	Does the journal accept different article types?	Yes
5	Does the journal use your full name or the correct name?	No
6	Does the journal copy many email addresses in "cc", "bcc" or "to" section of the email?	Yes
7	Do you receive such CFPs regularly from the journal?	Yes
8	Does the journal offer you a discount on APCs?	Yes
9	Does the journal mention your previous publication using your article title to show their interest in your research?	Yes
10	Does the journal invite you to join its editorial board, perhaps with a monetary incentive?	Yes

 Here are a few extra red flags related to website of a journal that may indicate that a journal is predatory:

 The journal charges exorbitant fees to publish an article but does not provide clear information about its editorial processes or the qualifications of its editorial board.

- The journal has a poorly designed website or no website at all.
- The journal has a high acceptance rate and claims to have a rapid review process.
- The journal has a vague or broad focus and accepts articles on a wide range of topics.
- The journal uses spam emails or other aggressive marketing tactics to solicit submissions.

By using a developed tool and lexicon, each CFP can be analyzed about this lexicon to understand its polarity and determine how likely the CFP might be from a possible predatory journal (RQ 4). There is no developed tool to encounter predatory journals, and current blacklist solutions suffer from weakness. Using the developed tool in current research will simplify detecting and distinguishing predatory or suspicious CFPs from legitimate ones. There is an important matter here that if a predatory journal uses the developed tool and enhances its CFP what will happen? In such a situation, a predatory journal has to remove many words that it uses to cheat authors. It has to be more honest (i.e., it cannot state that is indexed in misleading metrics)

## Conclusion

 The problem of predatory journals, also known as “predatory publishers,” is a significant and ongoing concern in the academic community. Predatory journals are publications that engage in unethical practices, such as charging authors substantial fees to publish their work without providing a proper peer review process or maintaining a reputable editorial board. These journals often solicit articles through calls for papers or emails and may use deceptive tactics to appear legitimate, such as using misleading or sensational subject lines, poor grammar and spelling, or fake or unfamiliar sender names and addresses.

 The proliferation of predatory journals has led to a number of problems, including the dissemination of flawed or unreliable information, the undermining of the credibility of legitimate research, and the waste of resources for authors who pay to have their work published in these journals. It has also contributed to a general mistrust of the academic publishing system and has made it more difficult for researchers to identify reputable journals in which to publish their work. Overall, the problem of predatory journals is a significant and ongoing concern in the academic community, and it is important for researchers to be aware of these unethical practices and to carefully evaluate the credibility of any journal before submitting their work to it.

 This study provides tips to help researchers identify CFPs from potential predatory journals that usually use similar wording and sometimes similar templates to encourage authors to submit papers. These flattering CFPs may be attractive to researchers unaware of the existence and behavior of predatory journals that commonly use accolades and positive wording to introduce themselves as prestigious publishers. Researchers should realize that all predatory journals are only interested in profit. They will be selfish and never state that they have low-quality or unexciting or nonexistent peer reviewing processes. Their indexing is usually forged and fictitious, and their editorial members seldom have the required qualifications. In other words, be reminded that “*Every cook praises his own broth*”.

 Overcoming the competition of publishing in recognized journals requires hard work and acceptance of critical reviews performed by qualified authors. Easy and fast publishing offered by predatory journals neither serves the authors nor the readers. It is only profitable to sham journal companies who show no respect for the amount of time spent by authors hoping to share their work and findings with members of scientific communities worldwide. The amount of discredit that predatory journals throw on scientific publishing should not be ignored and should be strongly fought by those to whom integrity and deontology are basic principles in life. To keep up to date on yet another related scam, we recommend reading about predatory conferences.^42^

 Our current research especially provides valuable insight but has some limitations. We only analyzed spam emails for a short duration. Future research can focus on long durations. A more detailed analysis of the various templates used by legitimate journals might detect significant differences. The present tool is an early version. Future research will improve our prototype. It should be noted that no list or tool removes the responsibility for researchers to educate themselves and carefully use their critical judgment to determine the quality of a journal. The terms in the lexicons require an update.

## Competing Interests

 There is no conflict of interest.

## Ethical Approval

 Not applicable.

## Supplementary Files


GitHub code: https://github.com/amrrs/spamming_detect_lexicon
Online tool: https://mdadkhah.shinyapps.io/PredatoryCFP/
