# Supplementary figures and images for: A Toolkit for Detecting Fallacious Calls for Papers from Potential Predatory Journals
Source: Adv Pharm Bull. 2023 Jan 23;13(4):627–34. doi: 10.34172/apb.2023.068 (PMC10676554; doi:10.34172/apb.2023.068)

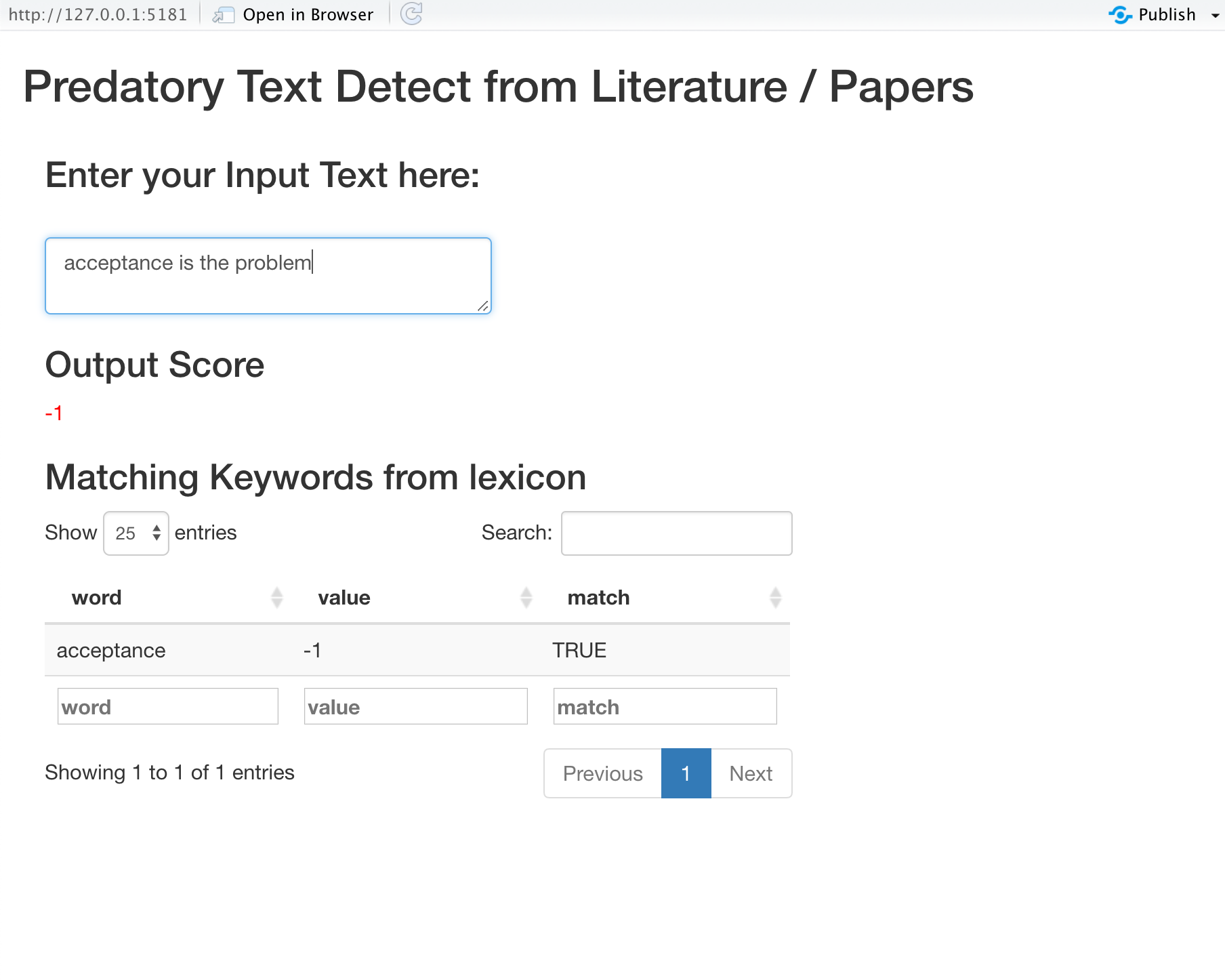

Supplement: Supplementary file 1 — GitHub code: https://github.com/amrrs/spamming_detect_lexicon Online tool: https://mdadkhah.shinyapps.io/PredatoryCFP/ [file apb-13-627-s001.zip › spamming_detect_lexicon-main/app_screenshot.png]
